# A computing platform for pairs-trading online implementation via a blended Kalman-HMM filtering approach

**DOI:** 10.1186/s40537-017-0106-3

**Published:** 2017-12-11

**Authors:** Anton Tenyakov, Rogemar Mamon

**Affiliations:** 1 Treasury Department, TD Bank Group, Toronto, ON Canada; 2 Department of Statistical and Actuarial Sciences, University of Western Ontario, 1151 Richmond Street, London, ON N6A 5B7 Canada

**Keywords:** algorithm fusion, investment, financial signal processing, change of measure, Ornstein–Uhlenbeck process

## Abstract

This paper addresses the problem of designing an efficient platform for pairs-trading implementation in real time. Capturing the stylised features of a spread process, i.e., the evolution of the differential between the returns from a pair of stocks, exhibiting a heavy-tailed mean-reverting process is also dealt with. Likewise, the optimal recovery of time-varying parameters in a return-spread model is tackled. It is important to solve such issues in an integrated manner to carry out the execution of trading strategies in a dynamic market environment. The Kalman and hidden Markov model (HMM) multi-regime dynamic filtering approaches are fused together to provide a powerful method for pairs-trading actualisation. Practitioners’ considerations are taken into account in the way the new filtering method is automated. The synthesis of the HMM’s expectation–maximisation algorithm and Kalman filtering procedure gives rise to a set of self-updating optimal parameter estimates. The method put forward in this paper is a hybridisation of signal-processing algorithms. It highlights the critical role and beneficial utility of data fusion methods. Its appropriateness and novelty support the advancements of accurate predictive analytics involving big financial data sets. The algorithm’s performance is tested on historical return spread between Coca-Cola and Pepsi Inc.’s equities. Through a back-testing trade, a hypothetical trader might earn a non-zero profit under the assumption of no transaction costs and bid-ask spreads. The method’s success is illustrated by a trading simulation. The findings from this work show that there is high potential to gain when the transaction fees are low, and an investor is able to benefit from the proposed interplay of the two filtering methods.

## Introduction

Pairs trading is an investment strategy used to exploit financial markets that are out of equilibrium. It consists of a long position in one security and a short position in another security at a predetermined ratio; see Elliott et al. [1]. Traders bet on the direction of the stocks relative to each other. Such a trading strategy is typically employed by hedge fund companies. Deviations in prices are monitored closely and used as basis for changing positions, taking advantage of market inefficiencies to obtain some profits. Hence, financial computing technologies with algorithmic trading capability, such as the one put forward in this paper, are necessary for the efficient and successful implementation of a pairs trade.

When the relative performance of the short position is better than that of the long position, this strategy is going to be profitable regardless of stock market regimes. So, a speculator who pairs a long position in a retail stock with a short position in a rival stock or an index ETF could end up with a profit. Pairs trading could be viewed alternatively as a mechanism to hedge both sector and market risks. When there is a financial crisis, two stocks both decline in value; but then the pair trade would result to a gain on the short position and an offsetting loss on the long position. This will yield a profit close to zero in spite of the large market movement. It is clear that this is a self-funding strategy as short sale proceeds may be utilised in creating the long position.

It is also worth mentioning that a pairs trade is essentially a mean-reverting strategy. That is traders bet that prices will revert to a long-run mean. Undoubtedly, the success of a pairs trading is mainly dependent on the modelling and forecasting of the returns spread between a pair of stock price processes. For this reason, this work promotes the use of a mean-reverting model hugely enhanced by the power of two filtering approaches combined together. Pairs trading is inherently built around models that support data mining and dynamic parameter estimation scheme. In this paper, a method featuring online algorithms is advanced, permitting quick buying and selling decisions that also give traders the capacity to take advantage of tighter spreads.

Elliott et al. [1] described two automated algorithms for setting a pairs-trading strategy. The strategy requires the support of a parameter estimation method. The first algorithm is based on the smoother approach (Shumway and Stoffer [2]), and the second algorithm is based on dynamic filtering in conjunction with the expectation–maximisation (EM) algorithm (Elliott and Krishnamurthy [3]). It was illustrated that both algorithms work rather well on simulated data with the latter showing a computational advantage. Unfortunately, due to the inherent formulation of Kalman filtering equations, the performance of the dynamic filtering algorithm is limited to models of white-noise type. Moreover, as Steele [4] pointed out, the model in Elliott et al. [1] has theoretical emphasis and does not delve into the important verification of the market’s stylised facts. For example, the normality of spread on equity returns (say, returns on a portfolio that is long on Dell stock and short on HP stock in equal dollar amounts) can be rejected, yet such a normality assumption is implicit in the posited model.

This paper contributes to the furtherance of techniques in stochastic modelling, information theory and soft computing technologies in the analysis of the financial system and transactions. It is aligned to the endeavours of creating tools for forecasting and investment strategies using hybrid techniques, and highlighting the potency of ensemble methods in the financial sector. For example, in stock-market prediction, Hassan et al. [5] combined HMM [6, 7] and fuzzy models.

In contrast, this research work integrates the HMM and Kalman algorithms primarily tailored to providing a support system for market-neutral trading. Consequently, the synthesis of these algorithms enables the processing of the spread data generated by two correlated stocks, and ultimately producing estimated optimal signals as critical inputs for executing trading rules or strategies. Filtering is promoted in conjunction with online data processing and intelligent data analysis supporting fact-based decision.

The remaining parts of this paper are structured as follows. "Background and related work" presents a brief literature review that features several algorithms currently employed in stock-market pairs trading. The contributions of this paper, and how it widens the set of available techniques are indicated in this section. "Modelling setup" details the modelling framework for the evolution of the underlying pairs or spread portfolio. In "HMM-extended Kalman filters", the construction of the filtering algorithms for the parameter estimation as well as the trading strategy is outlined. The effectiveness of the proposed approach is investigated in "Numerical demonstration" by setting trades based on simulated and historical data, where the spread process exhibits a strong non-normal behaviour. Some pertinent insights are given in “Concluding remarks”.

## Background and related work

A motivation of this work, within the context of big data (i.e., large financial data sets in real time), resembles to that of Cerchiello et al. [8] and Ouahilal et al. [9], which is the development and evaluation of trading models and systems to support investors. In this paper, the idea of an automated algorithm proposed by Elliott and Krishnamurthy [3] is extended under a model with non-normal noise. An HMM is used to drive the dynamics of model parameters; although the HMM in this discussion evolves in discrete time, it is also possible to modulate model parameters with an HMM in continuous time [10] with time-varying jump intensities. The HMM embedding in turn will enable the capturing of non-normality of returns [11], which is common in the spread portfolio. Lévy-type processes are noted to yield excellent statistical fit to the non-normal asset-returns process but they are difficult to interpret from a financial perspective. The novelty of the proposed approach lies on the modification and dynamic interplay of filtering algorithms due to Elliott and Krishnamurthy [3] and Erlwein and Mamon [12], and customised to support the implementation of trading strategies.

There are many examples demonstrating a markedly better fit to financial data when using an HMM compared to a simple autoregressive model. The method being put forward is more flexible than the Gaussian mean-reverting model, even for the generalised mean-reverting framework considered in Chen et al. [13, 14]. A mixture of normal models is employed and this features a couple of apparent advantages: (a) the modelling framework and methodology for the non-normal noise case depends only on a combination of already known and simple techniques for the normal case and (b) an easy economic interpretation could be put forward for the sudden changes in the behaviour of the process. From the practitioners’ point of view, the most desirable characteristic of the HMM-modulated model is its tractability for applications in the financial industry.

Although pairs trading is deemed relatively safe for market or sector arbitrageurs, nothing is really safe from the price swings within a sector. Such price swings could obliterate a short-term profit, and in some cases, bankrupt a financially stable hedger; see for example, Goldstein [15]. Typically, this risk is dealt with by unwinding a short position and attempting to absorb minimal losses. Such a strategy is taken by aggressive high-frequency algorithmic traders as their positions are usually neutral at the end of each trading day. Alternatively, as described in Khandani and Lo [16], a trader needs to monitor closely a constantly changing reverting level of the portfolio. This is because exit from an unprofitable trade is necessary when reversion to the mean does not materialise. This clearly implies that spread modelling and generating of forecast error bounds are critical as they provide the foundation of decision-making on when trade exits must occur.

The aim of this work complements that of [17], which uses a stochastic-control approach to pairs trading but with a numerical validation that relied on simulated data. In the case of this work, a method is developed that optimally and dynamically processes financial market signals thereby training model parameters so that they adapt well to market changes. This will precipitate a decrease in the size of the traders’ positions and consequently, will decrease potential risks as well. Akin to such an effect is the drawback that the hedger’s position, comprising of a short and a long leg within the spread, will also decrease the potential profit. This paper includes results from a numerical implementation that offer insights for a better understanding of how pairs trading works along with its financial implications. The integrated Kalman-HMM filtering algorithms could be interfaced with today’s computing technologies to support the successful pairs-trading implementation in the industry, which undoubtedly relies on the accurate modelling of the spread series.

## Modelling setup

In this section, the modelling framework is introduced by first defining the observation, state, and hidden-state processes. The trading process is then motivated and explained.

### Observed and hidden state processes

An Ornstein–Uhlenbeck (OU) process $r_t$ is a stochastic process that satisfies the Stochastic Differential Equation (SDE)

1 $$\begin{aligned} dr_t = b(\mu - r_t)dt + \sigma dW_t, \end{aligned}$$

where $W_t$ is a standard Brownian motion defined on a probability space $(\Omega , \mathcal{C}, P)$; $b,~\mu$ and $\sigma$ are positive constants independent of $W_t$. The parameter $\mu$ is the mean level to which the process tends to hover over time, *b* is the speed of mean reversion, and $\sigma$ is the volatility.

Of course, in reality, it is very rare that the OU parameters are constants as time *t* progresses. Thus, $b,~\mu$ and $\sigma$ will be made stochastic but their simplicity will be retained by letting them switch, at random time, between a finite number of values only. To attain this and enable the implementation of the discretised OU process, the parameters are assumed to depend on some underlying discrete-time Markov process $\{{\mathbf x}_k \}$. So, $b = b ({\mathbf x}_k), \mu = \mu ({\mathbf x}_k)$ and $\sigma = \sigma ({\mathbf x}_k)$ with

2 $$\begin{aligned} {\mathbf x}_{k+1} = {\varvec{\Pi }} {\mathbf x}_k + {\mathbf v}_{k+1}, \end{aligned}$$

where ${\varvec{\Pi }}$ and ${\mathbf v}_{k+1}$ are the respective transition probability matrix and martingale increment. That is, $E[{\mathbf v}_{k+1} | \mathcal {F}_k] = {\mathbf 0} \in \mathbb {R}^N$ and $\mathcal {F}_k = \sigma \{{\mathbf x}_0, {\mathbf x}_1, ... , {\mathbf x}_k\}$ is the filtration generated by $\{{\mathbf x}_k \}$. Let $\mathcal {C}_k$ be the filtration generated by $\{ r_k \}$. The states of ${\mathbf x}_k$ are mapped into the canonical basis of $\mathbb {R}^N$, which is the set of unit vectors ${\mathbf e}_h,~h=1, 2, \ldots , N$ with ${ {\mathbf e}_h = (0,~\ldots ,~1,~\ldots ,~0)^\top }$ and $\top$ is the transpose of a matrix. That is, the hth component of ${\mathbf e}_h$ is 1 and 0 elsewhere.

Following Erlwein and Mamon [12] or Tenyakov et al. [18], if the parameters in the solution of the OU process in Eq. (1) are HMM-driven then

3 $$\begin{aligned} r_{k+1} = \nu ({\mathbf x}_k) r_k+\zeta ({\mathbf x}_k)+\xi ({\mathbf x}_k) \omega _{k+1}, \end{aligned}$$

where

4 $$\begin{aligned} \nu ({\mathbf x}_k) = e^{-b({\mathbf x}_k) \Delta t}, ~~\zeta ({\mathbf x}_k) = (1- e^{-b({\mathbf x}_k) \Delta t})\mu ({\mathbf x}_k), \end{aligned}$$

5 $$\begin{aligned} \xi ({\mathbf x}_k)=\sigma ({\mathbf x}_k) \sqrt{\frac{1-e^{-2 b({\mathbf x}_k)\Delta t}}{2 b({\mathbf x}_k) }} ~~~~~~~~~~~~~~~~~~~~ \end{aligned}$$

and $\omega _{k+1}$ is a standard normal random variable. In Eqs. (4), (5), $\mu ({\mathbf x}_k)=\langle {\varvec{\mu }}_k, {\mathbf x}_k \rangle$, $b({\mathbf x}_k)=\langle {\mathbf b}_k, {\mathbf x}_k \rangle$ and $\sigma ({\mathbf x}_k)=\langle {\varvec{\sigma }}_k, {\mathbf x}_k \rangle$, where $\langle \cdot , \cdot \rangle$ is the usual scalar product. Such representation of the model parameters is the offshoot of having $\mathbb {R}^N$’s canonical basis as the Markov chain’s state space.

Adopting Elliott et al. [1], the observation process $y_k$ follows the state process ${r_k}$ observed in some Gaussian noise with a scaling coefficient $\alpha$. Thus,

6 $$\begin{aligned} y_k = r_k + \alpha Z_k, \end{aligned}$$

where $Z_k$ is a standard Gaussian random variable independent of $\mathcal {C}_k$.

To summarise, the equations, in discretised form, describing the dynamic behaviour of the data are

$$\begin{aligned}& {\mathbf x}_{k+1} = {\varvec{\Pi }} {\mathbf x}_k + {\mathbf v}_{k+1}~~\text{(hidden-state } \text{ process); } ~~~~~~~~ \\& r_{k+1} = \nu ({\mathbf x}_k) r_k +\zeta ({\mathbf x}_k)+\xi ({\mathbf x}_k) \omega _{k+1}~~\text{(state } \text{ process); }  \\& \text{ and }~~y_k= r_k + \alpha Z_k~~\text{(observation } \text{ process). } \end{aligned}$$

### The trading strategy

Define ${\mathcal Y}_k = \sigma \{y_0, y_1, ... , y_k\}$ as the filtration encapsulating the whole market information available up to time *k*. The ultimate goal is to compute the quantity $\widehat{r}_{k|k-1} = E[r_k|{\mathcal Y}_{k-1}].$ Write $\widehat{r}_{k|k-1}(i): = E[r_k|{\mathcal Y}_{k-1}, R_{k}=i],$ where $R_{k}=i$ represents the hidden ith state at time *k* of the Markov chain. The quantity $\widehat{r_k}(i)$ is interpreted as the most likely value of $r_k$ given the observed information up to time *k*.

Let $\theta _i$ be the unconditional probability of being in state *i*. The trading process is executed as follows: (a) find the values *i* and $i+1$ such that $\widehat{r}_{k|k-1}(i)<y_k<\widehat{r}_{k|k-1}(i+1)$; (b) aggregate the probabilities subject to $\Theta _1 = \sum _{j=1}^{i} \theta _j$ and $\Theta _2 = \sum _{j=i+1}^{N} \theta _j$; and (c) change the positions in a manner similar to that in Elliott et al. [1] with the key difference that the portfolio consists of two parts proportional to $\Theta _1$ and $\Theta _2$. The pairs-trading strategy is formed using two stocks with the predetermined ratio 1:1 as the stocks are taken from the same sector with a long position on one asset and a short in the other. The positions are set in the same way that the spread portfolio is either short or long depending on the expected level of the difference in the stock prices. If the level is lower than the spread then it is expected that the correction will occur so that the trader takes a long position on the spread portfolio, and vice versa.

**Fig. 1:**
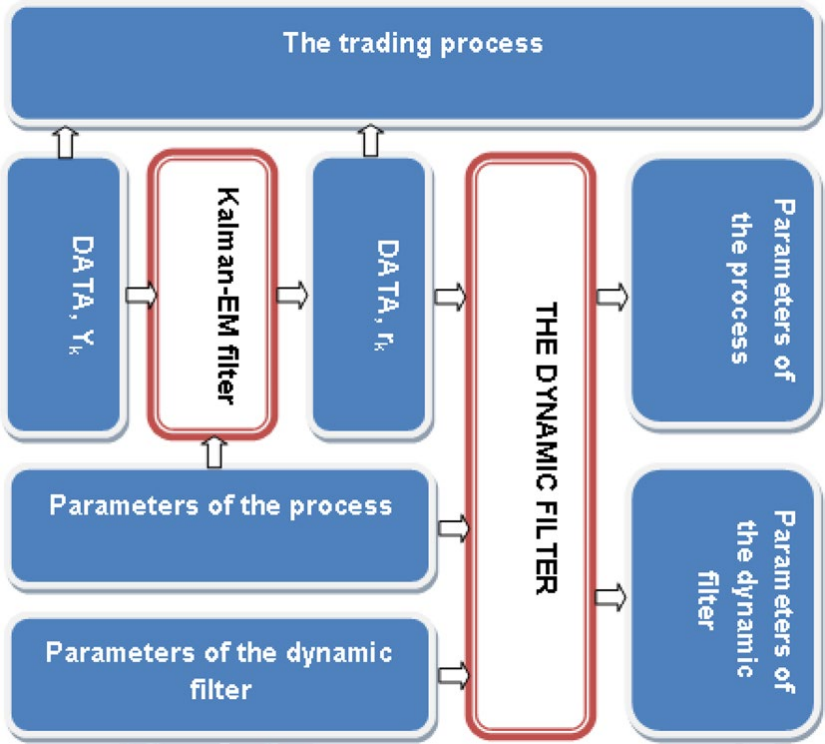
Schematic diagram in the implementation of the pairs-trading strategy

The graphical description of the trading process is displayed in Fig. 1. If $\text{ Ntnl }$ represents the total notional amount for the investment in the strategy, $\text{Ntnl}_l = \text{Ntnl}\Theta_l$ for $l=1,~2.$ The two trades are carried out simultaneously, i.e., $y_k<\widehat{r}_{k|k-1}(i+1)$ for $\text{ Ntnl }_2$ and $\widehat{r}_{k|k-1}(i)<y_k$ for $\text{ Ntnl }_1$. When $\widehat{r}_{k|k-1}(i)<y_k$, the spread is assumed to be too large so that a long position in the spread portfolio is taken. Similarly, when $y_k<\widehat{r}_{k|k-1}(i+1)$, a short trade is entered. This trading strategy leads to a substantial decrease in the overall risk exposure.

## HMM-extended Kalman filters

In this section, the Kalman filtering results, within the multi-regime set up, are provided. The dynamic filtering results for the OU process are briefly recalled as well.

### HMM-extended Kalman filter

The conditional mean and variance of the state process $r_k$ are derived below. The results are expressed in recursive relations. In particular,

7 $$\begin{aligned} \widehat{r}_{k|k-1}&=\sum _{i=1}^N\widehat{r}_{k|k-1}(i)\theta _i =\sum _{i=1}^N E[r_k|\mathscr {Y}_{k-1}, R_{k}=i] \theta _i =\sum _{i=1}^N E[\nu ({\mathbf e}_i) r_{k-1} +\zeta ({\mathbf e}_i)+\xi ({\mathbf e}_i) \omega _{k+1}|\mathscr {Y}_{k-1}, R_{k}=i] \theta _i \nonumber \\&=\sum _{i=1}^N E[\nu ({\mathbf e}_i) r_{k-1}+\zeta ({\mathbf e}_i)|\mathscr {Y}_{k-1}, R_{k}=i] \theta _i =\sum _{i=1}^N \left( \nu ({\mathbf e}_i) E[r_{k-1}|\mathscr {Y}_{k-1}, R_{k}=i]+\zeta ({\mathbf e}_i)\right) \theta _i \nonumber \\&=\sum _{i=1}^N \theta _i\nu ({\mathbf e}_i) E[r_{k-1}|\mathscr {Y}_{k-1}, R_{k}=i]+ \sum _{i=1}^N\zeta ({\mathbf e}_i) \theta _i =E[r_{k-1}|\mathscr {Y}_{k-1}]\sum _{i=1}^N \theta _i\nu ({\mathbf e}_i) +\sum _{i=1}^N\zeta ({\mathbf e}_i) \theta _i \nonumber \\&=\widehat{r}_{k-1}\widehat{\nu }+\widehat{\zeta }. \end{aligned}$$

Also,

8 $$\begin{aligned} \Sigma _{k+1|k}&=E \left[\left( r_{k+1} - \widehat{r}_{k+1}\right) ^2| \mathscr {Y}_{k}\right] =E\left[ \left( \nu ({\mathbf x}_k) r_{k}+\zeta ({\mathbf x}_k) +\xi ({\mathbf x}_k) \omega _{k+1} - \widehat{r}_{k+1}\right) ^2| \mathscr {Y}_{k}\right] \nonumber \\&=E\bigg [\bigg ( \nu ({\mathbf x}_k) r_{k}+\zeta ({\mathbf x}_k) + \xi ({\mathbf x}_k) \omega _{k+1} - \widehat{r}_{k}\widehat{\nu }-\widehat{\zeta } \bigg )^2| \mathscr {Y}_{k}\bigg ] =E\bigg [E\big [\left( \nu ({\mathbf x}_k) r_{k}+\xi ({\mathbf x}_k) \omega _{k+1} - \widehat{r}_{k}\widehat{\nu } \right) ^2| \mathscr {Y}_{k},R_{k+1} \big ]\bigg ] \nonumber \\&=E\left[E\left[\left( \nu ({\mathbf x}_k) r_{k} - \widehat{r}_{k}\widehat{\nu } \right) ^2| \mathscr {Y}_{k},R_{k+1} \right]\right] + E[\xi ^2 ] =E[\nu ^2]\Sigma _{k|k} + E[\xi ^2 ] =\widehat{\nu }^2\Sigma _{k|k} + \widehat{\xi }^2. \end{aligned}$$

The Kalman-updating formula is given by

9 $$\begin{aligned} \widehat{r}_{k+1} = \widehat{r}_{k+1|k} + M_{k+1}\left( y_{k+1} - \widehat{r}_{k+1|k}\right) , \end{aligned}$$

where $M_{k+1}$ is called the Kalman gain and determined in the state covariance estimation below. The conditional variance is calculated as

10 $$\begin{aligned}&E\left[\left( r_{k+1} - \widehat{r}_{k+1}\right) ^2|\mathscr {Y}_{k}\right] =E\left[\left( r_{k+1} - \widehat{r}_{k+1|k} - M_{k+1}[y_{k+1}-\widehat{r}_{k+1|k}]\right) ^2 |\mathscr {Y}_{k}\right] \nonumber \\&= E\left[\left( r_{k+1} - \widehat{r}_{k+1|k} -M_{k+1}[r_{k+1}+\alpha Z_{k+1}-\widehat{r}_{k+1|k}]\right) ^2 |\mathscr {Y}_{k}\right] = \left( 1-M_{k+1}\right) ^2 \Sigma _{k+1|k} +M_{k+1}^2 \alpha ^2. \end{aligned}$$

Minimising (10), the optimal value for $M_{k+1}$ is

11 $$\begin{aligned} M_{k+1} = \frac{\Sigma _{k+1|k}}{\Sigma _{k+1|k}+\alpha ^2}. \end{aligned}$$

Consequently, having obtained $M_{k+1}$ in (11), the updated state covariance is

12 $$\begin{aligned} \Sigma _{k+1|k+1} = \Sigma _{k+1|k} - M_{k+1}\Sigma _{k+1|k}, \end{aligned}$$

which reconciles with the result on p. 174 of Aggoun and Elliott [19] for Kalman filters with no regime switching.

Equations (7)–(12) exhibit semblance to those Kalman filter equations for the static case, i.e., when the parameters of the OU process are constants. If the set of parameters $\mathcal {S} =\{ \nu _i,~\zeta _i, ~\theta _i \,\, \text{ for }\,\, i=1, \ldots , N\}$ is provided, $\widehat{r}_{k+1|k}$ or $\widehat{r}_{k|k}$ can be found by applying Eqs. (7)–(12) recursively.

The sample standard deviation estimate is used to calculate $\widehat{\alpha }$ given by

13 $$\begin{aligned} \widehat{\alpha } = \sqrt{\frac{\sum _{j=1}^n \left( y_j - \widehat{r}_{j}\right) ^2}{n-1}}. \end{aligned}$$

### Parameter estimation

To improve modelling performance by enhancing model flexibility along with the provision of an accurate and efficient parameter estimation, the OU-HMM-based filtering method is applied to the Kalman-filtered data time series $\widehat{r}_k: = \widehat{r}_{k|k}$. All HMM filtering equations are established under the reference probability measure $\bar{P}$ and the results are reverted back to the real-world measure *P*. This is justified by the Bayes’ theorem and the associated Radon–Nikodym derivative involved in this measure change is given by

$$\begin{aligned} \Lambda _k= \frac{dP}{d\bar{P}}\bigg |_{\widehat{\mathscr {C}}_k} = \prod _{j=1}^k \lambda _j, \quad k\ge 1,\,\, \Lambda _0\equiv 1, \end{aligned}$$

where

$$\begin{aligned} ~2\ln \lambda _k=-\frac{\widehat{r}_k\left( \widehat{r}_{k-1}\nu ({\mathbf x}_{k-1}) + \zeta ({\mathbf x}_{k-1}) \right) - \left( \widehat{r}_{k-1} \nu ({\mathbf x}_{k-1})+ \zeta ({\mathbf x}_{k-1}) \right) ^2}{\xi ({\mathbf x}_{k-1})^2}, \end{aligned}$$

and $\widehat{\mathscr {C}}_k$ stands for the filtration of the process $\widehat{r}_k$ up to time *k*.

Write the conditional probability of ${\mathbf x}_k$ given $\widehat{\mathscr {C}}_k$ under *P* as

$$\begin{aligned} \beta _k^i:=P\left({\mathbf x}_k= {\mathbf e}_h | \widehat{\mathscr {C}}_k\right)=E\left[\langle {\mathbf x}_k, {\mathbf e}_h \rangle | \widehat{\mathscr {C}}_k\right]. \end{aligned}$$

and $$\widehat{\mathbf \beta }_k = \left(\widehat{\beta }_k^1, \widehat{\beta }_k^2, \ldots , \widehat{\beta }_k^N\right)^\top \in \mathbb {R}^N.$$ Now,

$$\begin{aligned} \widehat{\varvec{\beta }}_k=E[{\mathbf x}_k | \widehat{\mathscr {C}}_k]= \frac{\bar{E} [\Lambda _k {\mathbf x}_k|\widehat{\mathscr {C}}_k]}{\bar{E} [\Lambda _k |\widehat{\mathscr {C}}_k]} \end{aligned}$$

by the Bayes’ theorem for conditional expectation.

Set ${\mathbf c}_k=\bar{E} [\Lambda _k {\mathbf x}_k | \widehat{\mathscr {C}}_k]$ and notice that $\displaystyle \sum\nolimits _{i=1}^{N}\langle {\mathbf x}_k, {\mathbf e}_i\rangle =1$. So,

14 $$\begin{aligned} \sum _{i=1}^{N}\langle {\mathbf {c}}_k, {\mathbf {e}}_i \rangle = \sum _{i=1}^{N} \langle \bar{E}\left[ \Lambda _k {\mathbf {x}}_k| {\widehat{\mathcal {C}}}_k\right], {\mathbf e}_i \rangle = \bar{E} \left[ \left. \Lambda _k \sum _{i=1}^{N} \langle {\mathbf x}_k, {\mathbf e}_i \rangle \right| \widehat{\mathscr { C}}_k \right] = \bar{E}\left[ \Lambda _k | \widehat{\mathscr {C}}_k\right]. \end{aligned}$$

Therefore, Eq. (14) implies that

$$\begin{aligned} \widehat{\varvec{\beta }}_k=\frac{{\mathbf c}_k}{\sum _{i=1}^{N} \langle {\mathbf c}_k, {\mathbf e}_i \rangle }. \end{aligned}$$

Following Erlwein et al. [20] and Erlwein and Mamon [12], write

15 $$\begin{aligned} \mathcal {J}^{js}_{k+1} {\mathbf x}: = \sum ^{k+1}_{n=1}\langle {{\mathbf x}_{n-1}, {\mathbf e}_j} \rangle \langle {{\mathbf x}_{n}, {\mathbf e}_s} \rangle \end{aligned}$$

16 $$\begin{aligned} \mathcal {O}^{j}_{k+1} {\mathbf x}:= \sum ^{k+1}_{n=1}\langle {{\mathbf x}_{n}, {\mathbf e}_j} \rangle \end{aligned}$$

17 $$\begin{aligned} \mathcal {T}^{j}_{k+1}(f) {\mathbf x}:= \sum ^{k+1}_{n=1}\langle {{\mathbf x}_{n-1}, {\mathbf e}_j} \rangle f(\widehat{r}_n,) \quad 1\le j \le N. \end{aligned}$$

Equations (15) and (16) represent the number of jumps from ${\mathbf e}_j$ to ${\mathbf e}_s$, and the amount of time that ${\mathbf x}$ occupies the state ${\mathbf e}_j$ up to $k+1$, respectively. The quantity $\mathcal {T}^{j}_{k+1}(f)$ in (17) is an auxiliary process dependent on the function *f* with $f(r) = r$ or $r^2$ or ${r}_{k+1}{r}_k.$ To find more compact and efficient representations of the filtering equations, define the matrix ${\varvec{D}}(r_k)$ with elements $d_{ij}$ by

18 $$\begin{aligned} \left( d_{ij}(r_k) \right) = {\left\{ \begin{array}{ll} &{} \exp \left( -\frac{\widehat{r}_k\left( \widehat{r}_{k-1}\nu _i + \zeta _i \right) - \left( \widehat{r}_{k-1} \nu _i+ \zeta _i \right) ^2}{2\xi _i} \right) \, \, \text{ for } \,\, i=j\\ &{}0 \, \, \, \, \, \, \text{ otherwise. } \end{array}\right. } \end{aligned}$$

For any process $G_k$, denote the conditional expectation, under $\bar{P}$, of $\Lambda _k G_k$ by $\gamma (G)_k:=\bar{E}\left[\Lambda _k G_k | \widehat{\mathscr {C}}_k\right].$ The recursive filters for ${{\mathbf c}}_k$, $\gamma (\mathcal{{J}}^{j,i} {\mathbf x})_k$, $\gamma (\mathcal{{O}}^{i} {\mathbf x})_k$ and $\gamma (\mathcal{{T}}^{i} (f){\mathbf x})_k$ are provided in Theorem 1.

#### **Theorem 1**

*Let* ${{\mathbf D}}$ *be the matrix defined in (* 18 *)*.* Then*

19 $$\begin{aligned} {{\mathbf c}}_k = {\varvec{\Pi }}{{\mathbf D}} {{\mathbf c}}_{k-1} \end{aligned}$$

20 $$\begin{aligned} \gamma ({{J}}^{j,i} {\mathbf x})_k = {\varvec{\Pi }}{{\mathbf D}}(r_k) \gamma ({{J}}^{j,i} {\mathbf x})_{k-1} +\langle {\mathbf c}_{k-1}, {\mathbf e}_i\rangle \langle {\mathbf D}(r_k){\mathbf e}_i, {\mathbf e}_i \rangle \pi _{ji}{\mathbf e}_j \end{aligned}$$

21 $$\begin{aligned} \gamma ({{O}}^{i} {\mathbf x})_k = {\varvec{\Pi }}{\mathbf D}(r_k) \gamma ({{O}}^{i} {\mathbf x})_{k-1} +\langle {\mathbf c}_{k-1}, {\mathbf e}_i\rangle \langle {\mathbf D}(r_k){\mathbf e}_i, {\mathbf e}_i \rangle {\varvec{\Pi }}{\mathbf e}_i \end{aligned}$$

22 $$\begin{aligned} \gamma ({{T}}^{i} (f){\mathbf x})_k = {\varvec{\Pi }}{\mathbf D}(r_k) \gamma ({{T}}^{i} (f){\mathbf x})_{k-1} +\langle {\mathbf c}_{k-1}, {\mathbf e}_i\rangle \langle {\mathbf D}(r_k){\mathbf e}_i, {\mathbf e}_i \rangle f(r_k){\varvec{\Pi }}{\mathbf e}_i. \end{aligned}$$

#### *Proof*

The proofs follow similar idea to the derivations of the filtering equations in Erlwein and Mamon [12] or Date et al. [21]. $\square$

#### **Theorem 2**

*If the data set with components* $r_1,r_2, \ldots ,r_k$ *is drawn from the model described in Eq*.* (* 3 *)* *then the EM parameter estimates are*

23 $$\begin{aligned}&{\widehat{\pi }}_{ji}=\frac{\gamma \left( {{J}}^{j,i} \right) _k}{\gamma \left( {{O}}^{i}\right) _k} \end{aligned}$$

24 $$\begin{aligned}&{\widehat{\nu }}_i=\frac{\gamma \left( {{T}}^{i} \left( r_{k+1},r_{k} \right) \right) _k - \zeta _i\gamma \left( {{T}}^{i} \left( r \right) \right) _k}{\gamma \left( {{T}}^{i} \left( (r)^2 \right) \right) _k } \end{aligned}$$

25 $$\begin{aligned}&{\widehat{\zeta }}_i=\frac{\gamma \left( {{T}}^{i} \left( r \right) \right) _{k+1} - {\widehat{\nu }_i}\gamma \left( {{T}}^{i} \left( r \right) \right) _k}{\gamma \left( {{O}}^{i}\right) _k } \end{aligned}$$

26 $$\begin{aligned}&{\widehat{\xi }}_i =\frac{\gamma \left( {{T}}^{i} \left( r^2 \right) \right) _{k+1} + {\widehat{\nu }_i}^2 \gamma \left( {{T}}^{i} \left( r^2 \right) \right) _{k}+{\widehat{\zeta }_i}^2\gamma \left( {{O}}^{i}\right) _k }{\gamma \left( {{T}}^{i} \left( r^2 \right) \right) _k }\nonumber \\&~~~~~~~~-2 \frac{{\widehat{\nu }_i}\gamma \left( {{T}}^{i} \left( r_{k+1},r_{k} \right) \right) _k +{\widehat{\zeta }_i}\gamma \left( \mathcal{{T}}^{i} \left( r \right) \right) _{k+1}}{\gamma \left( {{O}}^{i}\right) _k } +\frac{{\widehat{\nu }_i}{\widehat{\zeta }_i}\gamma \left( {{T}}^{i} \left( r \right) \right) _k}{\gamma \left( {{O}}^{i}\right) _k }. \end{aligned}$$

#### *Proof*

The derivations of (23)–(26) are immediate from Erlwein and Mamon [12]. $\square$

The EM algorithm in Dempster et al. [22] is used to get the model parameter estimates. The estimates of $\nu ,~\zeta ~\text{ and }~ \xi$ can be obtained from Theorem 2, and their updates can be obtained by applying Theorem 1.

## Numerical demonstration

### Preliminary results

The integrated filters are applied first to simulated data. It will be shown that the new methodology adapts well not only during turbulent periods when the data display distinct multi-regime spikes in log-returns, but also during relatively calm times.

The OU process has a normal distribution and the increments $\displaystyle \frac{r_{k+1}-r_k}{r_k}$, conditional on $r_k$, are also normally distributed. From (3) and (6), the observation process $y_k$ can be expressed as $\displaystyle y_k = \nu r_{k-1} + \zeta + \xi \omega _k + \alpha Z_k,$ which is equivalent to

27 $$\begin{aligned} y_k {\mathop {=}\limits ^{\text{ dist }}} \nu r_{k-1} + \zeta + \sqrt{\xi ^2 + \alpha ^2} \widehat{\omega _k}. \end{aligned}$$

Clearly, Eq. (27) has an OU functional form too, where $\widehat{\omega }_k$ is distributed as *N*(0, 1). Unfortunately, real data in practice often do not satisfy the normality hypothesis. A Markov-driven model is robust in capturing various types of data distributions observed financial data. Such a model also has the potential to be employed extensively for long periods characterised by a mixture of calm and turbulent financial times.

For simulation and benchmarking intents, a simplified version of (3) is considered, without regime-switching, according to the equations

$$\begin{aligned} r_{k+1} = 0.6 r_k+ 0.15 + 0.05 \omega _{k+1}~~\text{ and }~~ y_k = r_k+ 0.4 Z_k. \end{aligned}$$

**Fig. 2:**
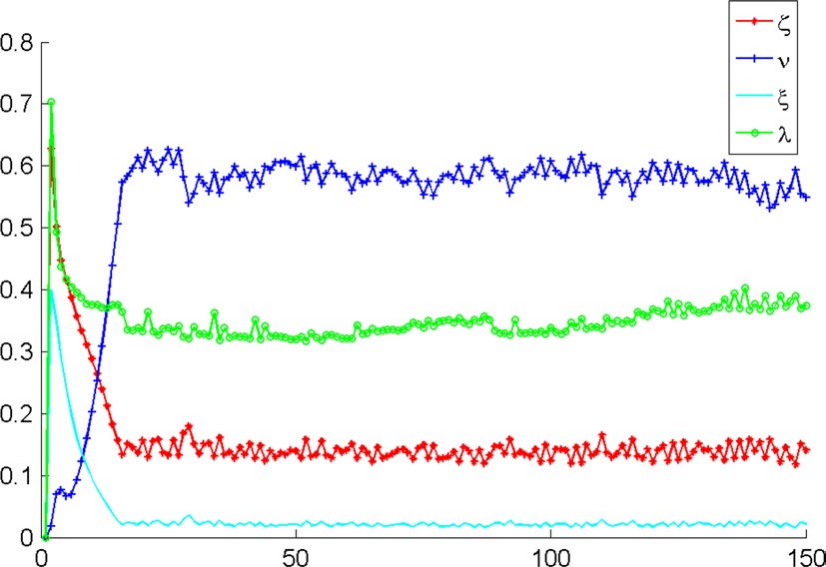
Single-regime dynamic filtered parameter estimates using simulated data based on ("Preliminary results")

From Fig. 2, it could be concluded that the filters of Elliott and Krishnamurthy [3] produce quite noisy but precise parameter values. The estimates are fairly stable and will not cause a significant portfolio loss if one decides to use them for financial market trading.

**Fig. 3:**
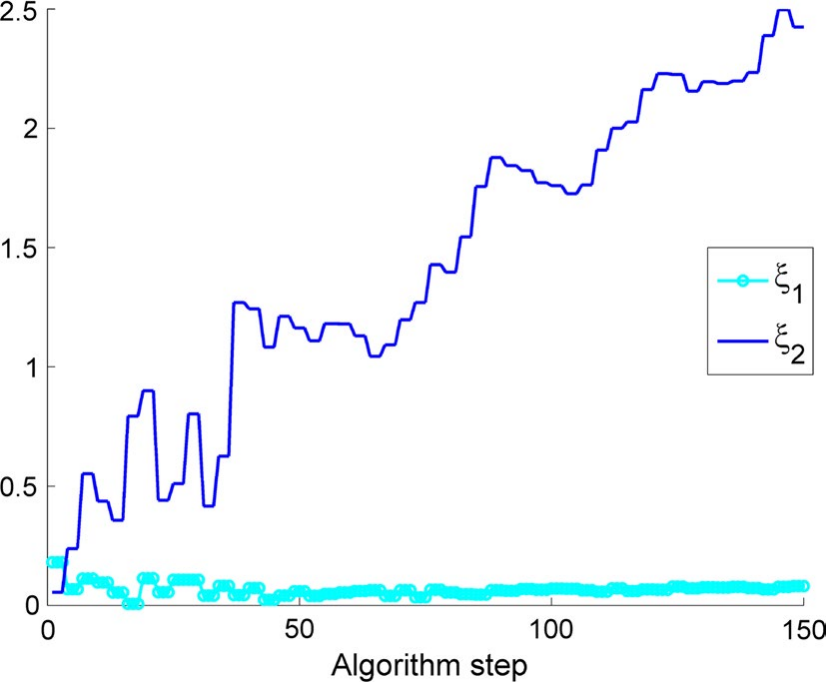
Evolution of the estimated $\xi _1$ and $\xi _2$ under a two-regime HMM

The proposed method and algorithm are assessed with respect to speed and relative error in the estimated EM estimates and other features of the algorithm. The dynamics of the parameters are estimated using the combined filters under the two-state model; the dynamic movement of $\xi$’s estimates are displayed in Fig. 3. Notwithstanding the algorithm’s main purpose, which is to replicate the dynamics of the coefficients modulated by the HMM, it does also estimate the one-regime (stationary) parameters very accurately. In particular, it only takes about half the time in obtaining the parameter estimates when the computation is compared to that under the Kalman-EM algorithm of Elliott and Krisnamurthy [3].

In contrast to the results depicted in Fig. 2, the filtered parameters from the proposed approach have better precision and flexibility than those given by the Kalman-EM filters. The increase in the computational time can be explained by the numerical implementation of the new method’s structure. Comprehensive details regarding the implementation of the multi-regime filter procedure can be found further in Tenyakov et al. [18, 23], and Erlwein et al. [20].

Although the filters in [18] and [23] are HMM-based, they do not fuse Kalman filters as in the case of this paper and their applications are also different. In particular, a one-step delay set up is considered in [18] with application to liquidity risk modelling and forecasting whilst a zero-delay framework is examined in [23] with application to high-frequency foreign exchange rates data.

### Data analysis

**Fig. 4:**
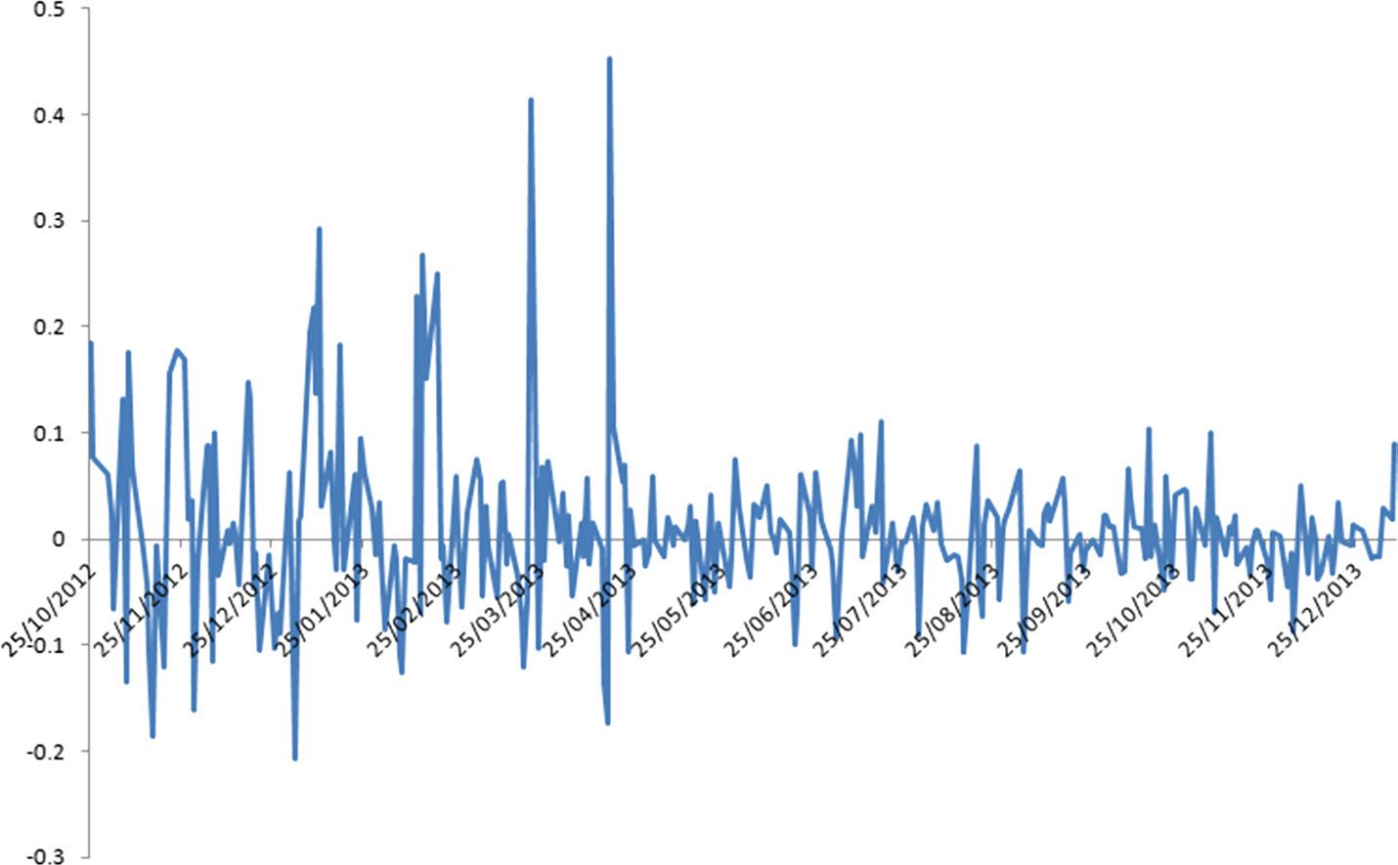
Spikes in the log of price spreads

By construction, the Kalman-EM filter (cf. Elliott et al. [1]) is not able to capture all stylised characteristics of the data governed by a non-normal distribution. Thus, this presents difficulty when implementing trading strategies based on Gaussian models. The KO–PEP (Coca-Cola Co–PepsiCo Inc) pair of stocks was chosen to test the integrated filtering approach put forward in this paper. The data on daily NYSE closing prices were downloaded from Bloomberg covering the period 24 October 2012–07 January 2014. Daily closing price data were used in this study as intra-day prices have additional liquidity and high-frequency-type noise is embedded in them. It is also known that intra-day data are observed in uneven intervals making them not straightforward for immediate analysis. The data set has 300 data points, and contains subsets with price spreads exhibiting distinct non-Gaussian spikes and possessing multi-regime behaviour; see Fig. 4.

The choice of KO and PEP in the implementation part of this paper stems from the fact that the two are premier global brands in the beverage sector. They have been around for more than a century, and marketing products worth billions of dollars yearly. KO and PEP have robust balance sheets but a lack of growth for both companies is somewhat concerning. The decrease in soda sales due to vigorous health consciousness campaigns in recent decades also affect the KO and PEP’s main lines of business. However, both companies are in competition as well in developing and growing a whole variety of new product innovations in aggressive efforts to replace unrealised and lost revenue. All of these homologous attributes make KO-PEP ideal as a pairs-trading pick.

Of course, the beverage stock pair (KO-PEP) chosen in this study is just a representative for the purpose of numerical illustration. As the proposed method is data-independent, it can be applied in general by a trader who would select other pairs of stock-market data with similar volatility and that are typically highly correlated. The modified spread (SPR) between KO and PEP, set as $\text{ SPR } = \text{ KO } - \text{ PEP } - 30,$ is used in the filtering procedure. The adjustment coefficient of 30 was chosen solely for convenience of representation. For the real trading procedure, the spread $\text{ KO }-\text{ PEP }$ is used. Note, that even with 30 as an adjustment, this does not result to negative values in the differences and the main characteristic of the data set is preserved. An attempt was made to find the rationale for the major spikes in $\text{ SPR }$. No explanation, however, from data sources such as Bloomberg and the Internet can be found. The occurrence of spikes appear data-specific, and looking at the same time series of spread from 2002, the spikes happened again quite frequently.

### Initialisation of the algorithm

To implement the filtering algorithms, suitable parameters for initialisation are needed. An advantage of the proposed approach is the complete automation of the initialisation stage. The financial modeller may choose from various algorithms to determine the initial parameters, some of which are described in Erlwein et al. [24], Date and Ponomareva [25], and Date and Bustreo [26], amongst others. For the purpose of this study, a simple log-likelihood maximisation would suffice and the procedure in Date and Ponomareva [25] is adopted; this is also described in Hardy [27]. The data set is divided into two parts: one part as a training subset, and the remaining part is employed for the trading procedure and validation.

To carry out the initialisation step, data set is assumed to follow the simple OU model, and so $\alpha =0$ in (6), and the other parameters are not modulated by a Markov chain. The likelihood function is then given by

$$\begin{aligned} L = \prod _{i=1}^{N_{trn}} \frac{1}{\sqrt{2\pi } \xi } \exp \left( {-\frac{({r}_{i+1} - \nu {r}_{i} - \zeta )^2}{2\xi ^2}}\right) \end{aligned}$$

or

28 $$\begin{aligned} L = \prod _{i=1}^{N_{trn}} \phi _{i}, \end{aligned}$$

where

$$\begin{aligned} \phi _{i} = \phi \left( \frac{r_{i+1} - \nu r_{i} - \zeta }{\xi }\right) \end{aligned}$$

and $\phi$ is the probability density function of a standard normal random variable.

The MLEs resulting from Eq. (28) are chosen as starting values for the estimation and trading procedure detailed in the later sections. To choose the optimal number of regimes, several model selection criteria could be employed such as the Akaike information criterion [28], C-hull criterion [29], and the Bayes information criterion [30]. Typically, the optimal number of regimes based on these criteria is two. The “curse of dimensionality” is still bearable at this level. In the new approach, the number of parameters will more than double if the number of regimes is increased to three. When the data set does not show extreme spikes (i.e., about 3 standard deviations or more from the mean), any increase in the number of regimes will expectedly result to accurate prediction of spread levels (this is called overfitting), however such setting is also penalised for its complexity.

The development of a filtering algorithm under a one-regime model is not considered here as this was already pursued in Elliott et al. [1]. Notwithstanding, this one-regime model is still used to benchmark the results of the suggested method. The initial estimates for the parameters of the filters are shown in Table 1.

**Table 1:** Initial parameter estimates for the multi-regime filtering algorithm

$\nu$	$\zeta$	$\xi$	$\pi _{1}$
0.9682	0.1225	0.2596	0.5

The same values are used for all regimes (e.g., $\nu =\nu _1=\nu _2$, etc.)

Following Elliott et al. [1] on the appropriate bounds of the parameter values (i.e., $0<\nu <1$, $\zeta >0$ as well as $\xi >0$), it could be concluded that the data set is suitably modelled by the OU process. The same values were used to initialise the benchmark algorithm. Nonetheless, in Elliott et al. [1] or Elliott and Krishnamurthy [3], there is no apparent indication on how to select the starting values. The convergence of estimates is expected as long as the starting values in the initialisation stage are reasonably close to the actual model parameters. In this study, it turns out that Elliott–Krisnamurthy dynamic filters do not produce any convergence and hence, parameters are adjusted to produce stable convergence on the training data set. The “stabilised” parameters are shown in Table 2.

**Table 2:** Initial parameter estimates used in applying the dynamic filtering algorithm of Elliott and Krishnamurthy [3]

$\nu$	$\zeta$	$\xi$
0.8731	1.5527	0.1364

### Testing on real data

In this section, the trading method is outlined in conjunction with the filtering algorithms. This procedure is performed in several steps. The optimal parameters are found using the EM algorithm. Then, $\widehat{r}_t$ is estimated and compared to the observed $y_t$. The comparison results are then used as inputs to establish the trading position.

Before applying the filters to the data, the user may decide if smoothing the data without introducing noise to the underlying process, is needed taking into consideration additional work and time for “cleaning” and smoothing. This is relevant to high-frequency data but not necessary for the purpose of this numerical experiment. Another important question is the length of data series to be included in one pass of the algorithm. Some insights on this issue are given in Erlwein et al. [20].

**Fig. 5:**
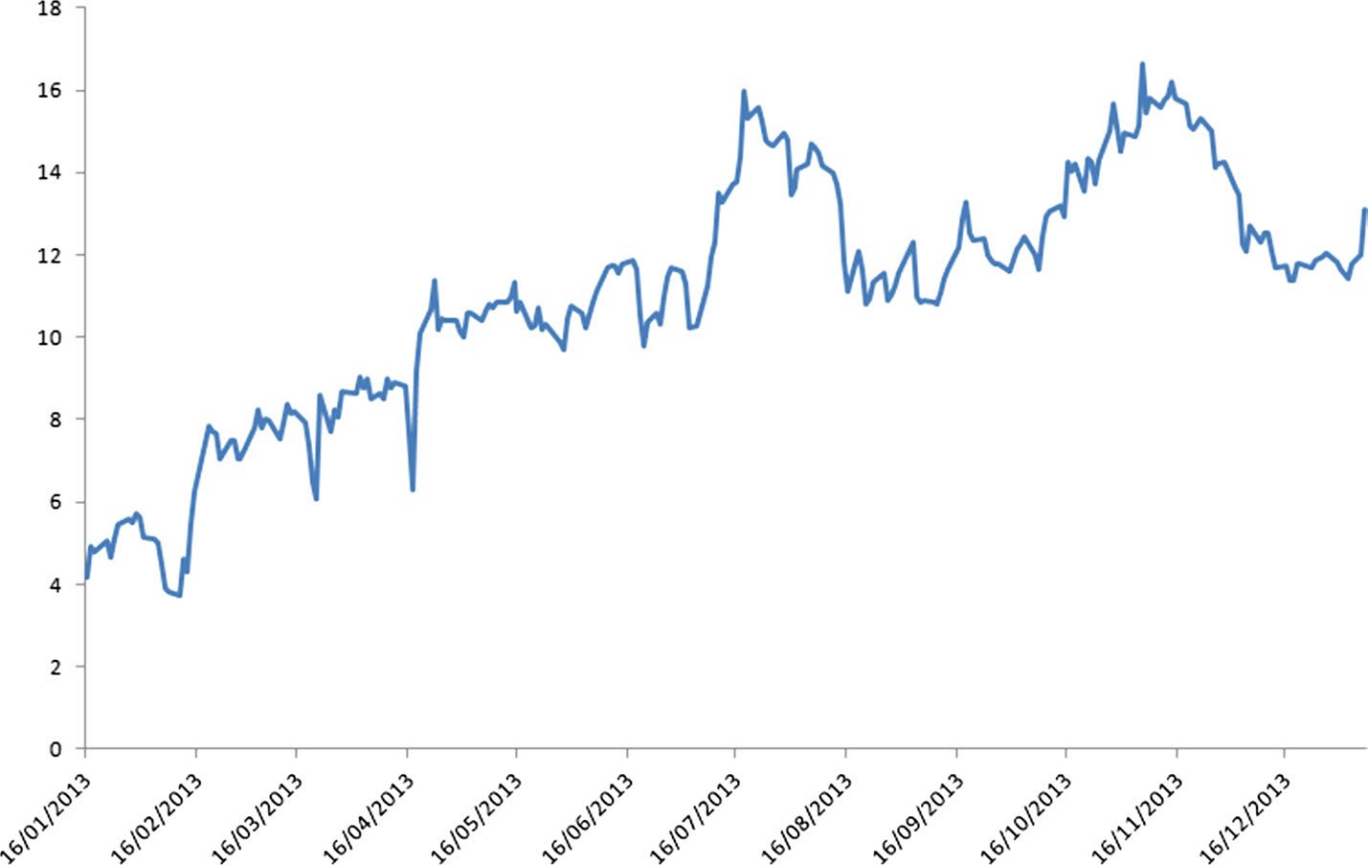
Dynamics of the spread $\text{ SPR }$ in the data subset used for parameter estimation

The new algorithm (combining the Kalman and multi-regime filters) is implemented with starting parameters given in Table 1, which were estimated using the data subset from 16 January 2013 to 07 January 2014 with a moving filtering window of three points. By taking a “practical” look at the data plotted on Fig. 5, it seems that the process produces 2–3 major and approximately 10–15 minor jumps every 50 points. The time series data do not appear to have a constant mean-reverting level. But, some stabilisation in the mean seems possible in the horizon. If the processing window of $50/15 \approx 3.33$ or bigger, the minor changes in the data set’s behaviour may be missed by the filter. This selection judgement on the window processing size is supported by the numerical work in Erlwein et al. [20]. The first 50 points of the data were “cleaned up” (i.e., processed using the Kalman estimation algorithm) and then the dynamic multi-regime filters are applied to the Kalman-filtered estimates to produce the parameters of the proposed hybrid algorithm.

The next step in the new algorithm is different from that in the pairs-trading paper of Elliott et al. [1]. For emphasis, the following shift happens not by one point, but by the size of the moving window which was established to be three. The long shift might affect two possible position changes between the first and third value. The step is repeated until all the data points are completely processed.

**Fig. 6:**
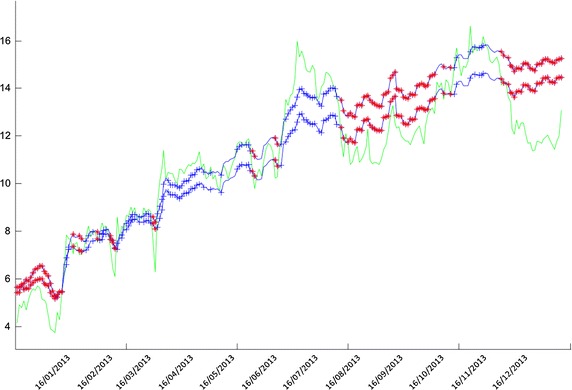
Data processed via the dynamic filtering algorithm

The plot of the estimated values of the spread in each state under the two-regime model is depicted in Fig. 6. The positions are also plotted, where the estimates from both regimes, $\widehat{r}_t(1)$ and $\widehat{r}_t(2)$, are either greater or smaller than the corresponding value for $r_t$. Points marked with $*$ indicate $\widehat{r}_t(i)>r_t$ for all *i*, and with $+$ indicate the reverse inequality relation.

Ostensibly, the predicted values of $\widehat{r}_t$ follow the dynamics of the process very well. Since a one-regime model is chosen to begin with, it takes some some time for the algorithm to show relative stability in the spread process signifying further support for the two-regime behaviour of the data. Questions about fitness and model error must be settled using some statistical methods and criteria. Since various model settings are developed for financial trading purpose in this paper, profit will be the main criterion for choosing the best model.

Four trading strategies are considered: (a) aggressive trade in which all earned extra capital is being reinvested in the stock; (b) normal trade in which only one unit of the stock is kept and all earned capital is invested in the money market; (c) safe trade is a variation of the normal trade and employed when there is uncertainty in the positions, i.e., $\widehat{r}_t(1)<r_t<\widehat{r}_t(2)$, in which case all the capital is invested in the money market; and finally, (d) imaginary trade in which the normal trade is performed on every data point of the “processed” data subset as described above; of course, this type of data set is not available in real time, and therefore, the result of this trade is used only for comparison.

The results in Table 3 show that the aggressive strategy does not necessarily produce a significant increase in profits. In investing all earned capital in the spread portfolio, the main part of the profit is collected whilst the investor takes a long position on the portfolio. Considering the quite long investment horizon, the increase in the value of the spread portfolio may be deemed not that significant. Still, it has to be noted that the overall profit is substantially greater than zero, and will be magnified depending on the beginning amount of the investment.

**Table 3:** Pairs-trading profits using the dynamic approach with interest rate of $0.01\%$/per day and initial capital of zero

	(a) Aggressive	(b) Normal	(c) Safe	(d) Imaginary
Profit ($)	16.6361	16.0067	15.8564	18.4892

A decrease in the portfolio value is apparently due to losses incurred during the “incorrect” trades. A decline in the profit is minimised by keeping a double position as explained in "The trading strategy". The minimal difference in the trading results between the safe and normal positions can be explained by the behaviour of $\theta _t$. If $\theta _t$ is always close to 0.5, this strategy corresponds to taking long and short positions with almost equal proportions of the portfolio. This means that the best strategy is doing nothing whilst investing all previous profits in the money market account.

As expected, the imaginary strategy outperforms all other trading strategies although the earned margin profit is not as high as one would predict. This arises from the size of the moving window, which sets the maximum profit strategy. If the frequency of changing positions increases, it is hard to say anything about the amount that will be earned or lost as a consequence of the frequent trade. Any extra profit or loss is treated as a result of pure noise and it cannot be controlled within the set up of the proposed approach.

**Fig. 7:**
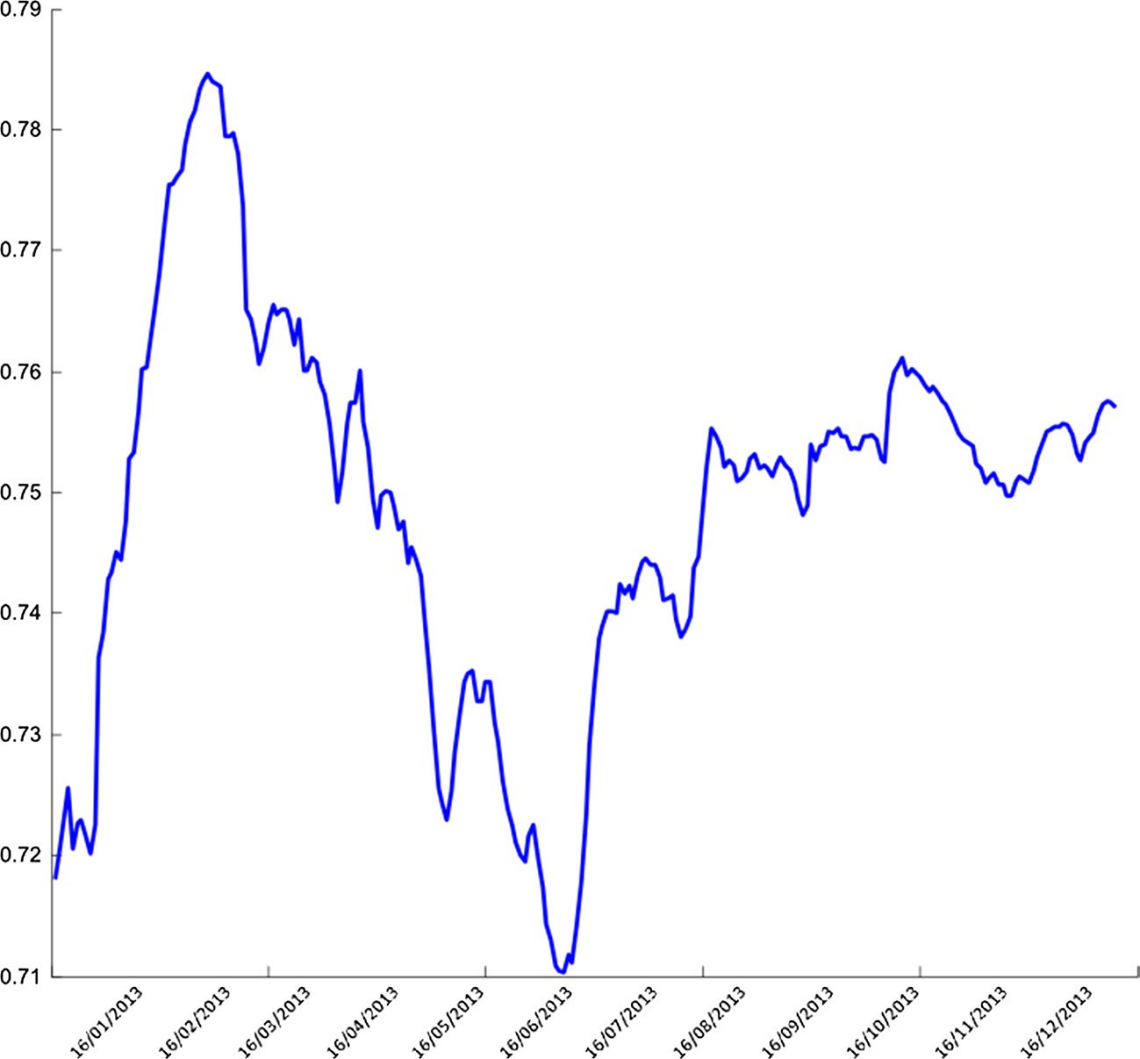
Evolution of the implied $\zeta$

**Fig. 8:**
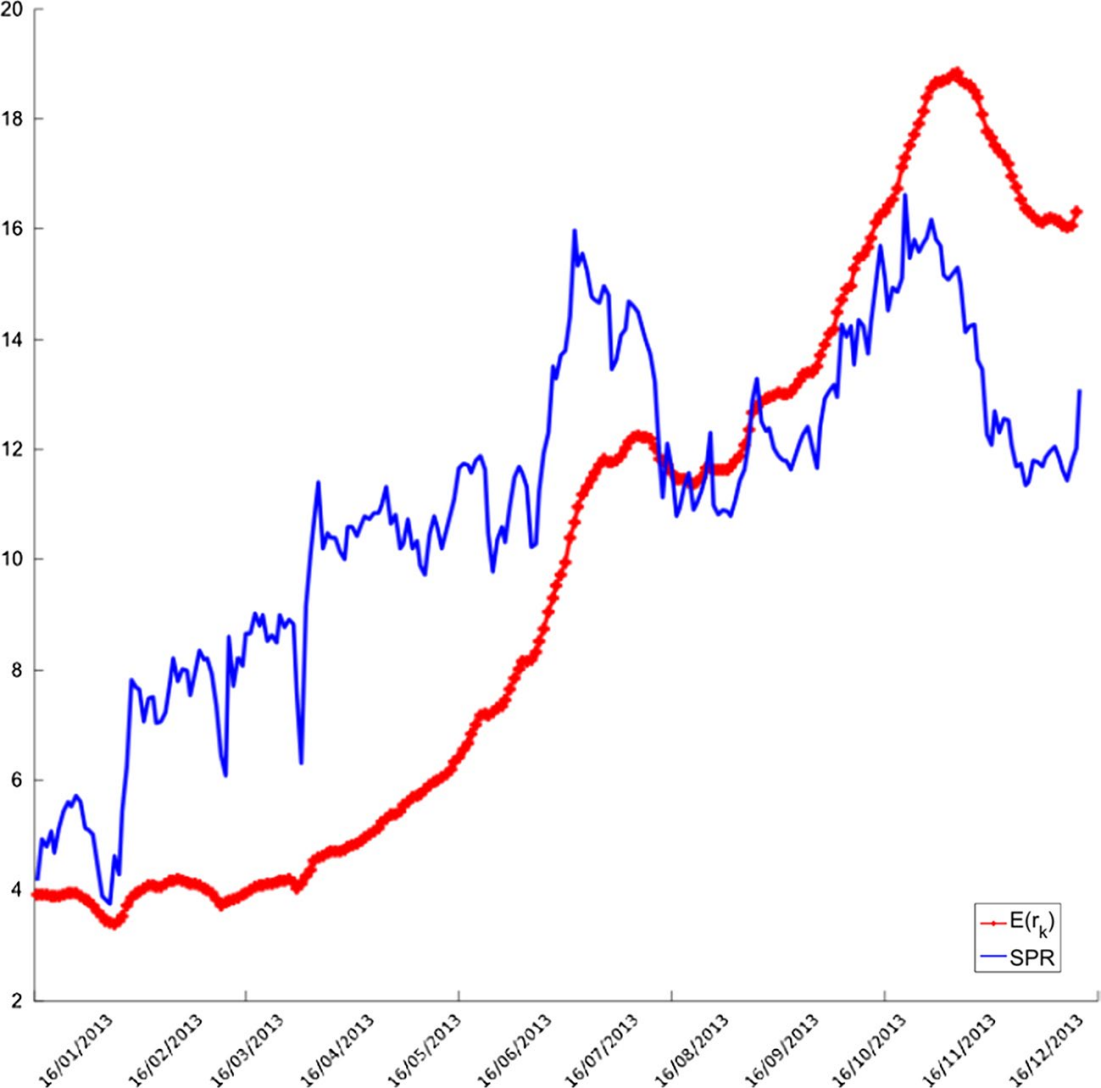
Comparison between $\widehat{r}_k$ and *SPR*

Using the initial values in Table 2, the parameters are estimated in the proposed modelling framework. The dynamics of the implied parameters $\nu ,~\zeta$, $\alpha$ and $\xi$ were calculated using the single-regime dynamic approach; the evolution for the estimated $\zeta$ is shown in Fig. 7. Figure 8 presents the graph of the “best” estimates of the process defined as $E[r_{k+1}] = \widehat{\nu } E[r_k|Y_k]+\widehat{\zeta }$.

Different sets of parameters were tried as starting values, but some divergence in the parameters’ behaviour does not disappear. This is to be expected anyhow because the data set dictates the dynamics of the parameters. If convergence has to occur, almost any set of values can be assumed as starting values for the algorithms.

## Concluding remarks

This work improved the performance of the pairs-trading strategy proposed by Elliott et al. [1]. The adaptive power of two popular filtering approaches, namely, the Kalman and HMM filters were blended together. Based on empirical evidence, the new hybrid algorithm outperforms each individual filtering technique. The proposed algorithm is more robust and well-suited to the pairs-trading strategy, and requires the same computational resources as those for the conventional Kalman filters on Gaussian-type data sets.

The extent of the gain’s significance in having multi-dimensional filters is explored with respect to accuracy without diminishing execution time and increasing complexity of the optimisation methods. Applications demonstrated in this paper proved successful in trading simulation. On the assumption of no transaction costs and data on bid-ask spreads, the back-testing trade performance showed that a hypothetical trader could manage to earn a non-zero profit with positive probability. The trading technique has the potential to yield positive gain under reasonable transaction fees. Although the approach based on the combination of two filtering methods were tested on historical prices of Coca-Cola Co and PepsiCo Inc, practitioners could adopt this to other pairs of data sets and exploit arbitrage opportunities due to temporary market inefficiencies or other factors.

The inclusion of transaction costs (e.g., [31]) for pairs trading under the multi-regime framework, and for instance when $r_k$ is not necessarily OU (e.g., [32, 33]) or OU but with additional statistical properties (e.g., [34]) is open for further investigation. The cost structure needs to be defined and embedded into the procedure. Also, the use of high-frequency data (e.g., [35]) may be considered, given the current practice of hedge funds companies and other institutional investors. Further examination is required on how to produce filtered parameter estimates within their specified constraints without, or at least with minimal, additional numerical optimisation procedures.
